# A fully water coupled oblique light-sheet microscope

**DOI:** 10.1038/s41598-022-09975-3

**Published:** 2022-04-08

**Authors:** Yiyang Gong, Yuqi Tian, Casey Baker

**Affiliations:** Department of Biomedical Engineering, Duke University, Durham, NC 27708 USA

**Keywords:** Neural circuits, Fluorescence imaging, Light-sheet microscopy

## Abstract

Recently developed descanned versions of the oblique light-sheet microscope promise to enable high-frame rate volumetric imaging in a variety of convenient preparations. The efficiency of these microscopes depends on the implementation of the objective coupling that turns the intermediate imaging plane. In this work, we developed a fully immersed coupling strategy between the middle and end objectives of the oblique light-sheet microscope to enable facile alignment and high efficiency coupling. Our design outperformed conventional designs that used only air objectives in resolution and light-collection power. We further demonstrated our design’s ability to capture large fields-of-view when paired with a camera with built-in electronic binning. We simultaneously imaged the forebrain and hindbrain of larval zebrafish and found clusters of activity localized to each region of the brain.

## Introduction

The exploration of the structure and function of biological tissue over millisecond- to hour-long timescales employs fluorescent imaging of labeled biological samples. Engineering of fluorescence optical imaging technologies are abound, each targeting a subset of biological samples with specific cell density, thickness, field- or depth-of-view, and scattering properties^1^. Biological samples with relatively high optical transparency and low scattering can be imaged by the family of light-sheet microscopes or selective plane illumination microscopes^2^. These microscopes scan one plane of excitation through the sample, and then descan the excitation plane to a fixed detector for imaging using electronically synchronized translation of optics or tuning of electrically tunable lenses^3^. The speed, resolution, and efficiency of these microscopes continue to rise, as they can attain sub-cellular resolution with millisecond temporal resolution.

The oblique illumination microscope^4^ or swept confocally-aligned planar excitation (SCAPE) microscope^5^ in the light-sheet family serves the specific application of imaging biological samples with access from only one direction. While the traditional light-sheet design excites samples through one objective and images the plane through a second orthogonally placed objective, the oblique design illuminates the sample with a tilted light-sheet and images the excitation plane through the same primary objective. The microscope then forms a tilted intermediate image with a secondary objective in a remote focusing configuration. Finally, an imaging path starting with a tertiary objective at an angle with respect to the secondary objective images the tilted intermediate image plane to a fixed sensor plane. The initial oblique light-sheet implementation produced volumetric recording by mechanically scanning the sample or mechanically scanning the intermediate objective^6^. More recent designs further improved the volumetric frame rate by scanning the excitation plane through the sample with a small galvo scanner, and descanning the excitation plane to the fixed sensor plane using the same scanner^7,8^ or a paired surface on a polygon mirror^5^.

Although the SCAPE design already has multiple implementations, portions of the microscope are still being engineered. One portion is the coupling between the secondary and tertiary objectives, which is a bottleneck in the transmission of the microscope. Past research used a variety of air objectives within this coupling^8^, as well as a variety of immersion-based attachments on objective 3 to increase the light capture power^9–11^. In this work, we design, implement, and characterize a fully water-immersed coupling between the last two objectives of the microscope. This coupling supported the use of high numerical aperture (NA) water immersion objectives in the turn of the SCAPE design. The high NA objectives in turn supported a large overlap between light cones within the oblique turn, and thus supported high transmission efficiency through the SCAPE pathway and high imaging resolution. We demonstrated such imaging capabilities by imaging fluorescent beads, pollen grains, and awake larval zebrafish.

## Results

### A rotated SCAPE design supported water immersion coupling between objectives

**Figure 1:**
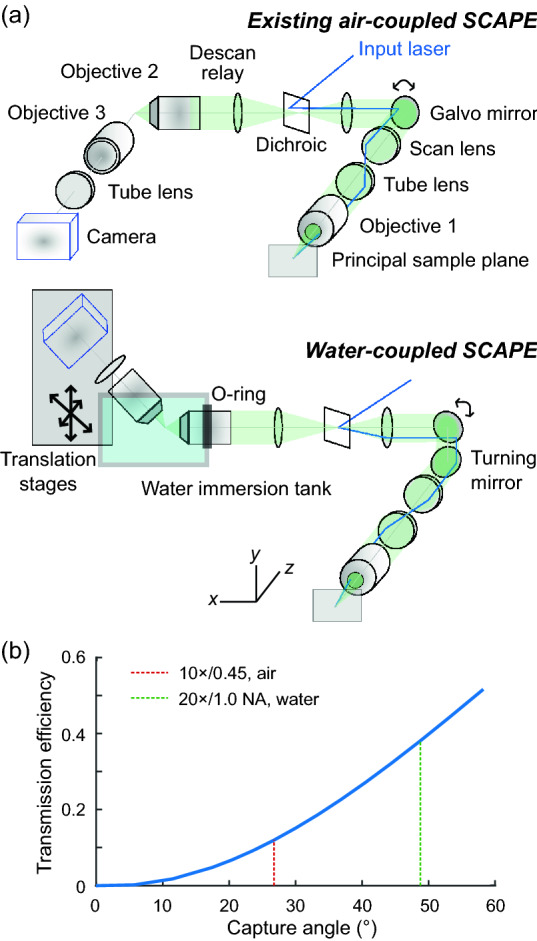
A water immersion system coupled two water immersion objectives and increased the SCAPE transmission efficiency. (**a**) *Top*: Conventional SCAPE scans an excitation oblique light sheet through the sample with a galvo mirror. It then descanned emission from the light-sheet through the same galvo mirror. It finally used a pair of air objectives, objective 2 and 3, to rotate the oblique light sheet to a fixed plane on the camera. *Bottom*: Water-coupled SCAPE employs the same principles as conventional SCAPE, but employs two high NA water immersion objectives as objectives 2 and 3. The panel labels highlight critical design differences between conventional SCAPE and water-coupled SCAPE: a water immersion tank coupled the two objectives, with objective 2 sealed in place with an O-ring; the imaging pathway starting with objective 3 was freely able to move via multiple translation stages and align to the intermediate image after objective 2; a turning mirror after the galvo mirror in the excitation pathway matched the excitation plane to the imaging plane defined by the objective 2-to-objective 3 coupling direction. (**b**) We calculated the transmission efficiency in the emission path from the objective 2 back aperture to the objective 3 back aperture as a function of the capture angle of objective 3. We fixed the oblique angle between the two objectives to 38°, and the objective 2 capture angle to 48°, representative of both a 0.75 NA air objective and a 1.0 NA water objective. We highlight the two objectives used for objective 3 in this work, the 0.45 NA air objective (*red*) and 1.0 NA water objective (*green*), with vertical dashes.

Existing SCAPE designs, which we call “conventional SCAPE” in this work, constructed the optical pathway primarily parallel to the table surface (Fig. 1a, *top*). The microscope directed the input laser away from the center of the optical pathway to generate an oblique excitation plane. A galvo mirror in the Fourier space of the microscope both scanned the excitation plane in the sample space and descanned the excitation plane to a fixed, oblique imaging plane behind the intermediate objective 2. The final imaging path consisting of objective 3, a tube lens, and a camera was rotated to match the angle of the oblique intermediate plane and image its contents.

The theoretical transmission efficiency of SCAPE from the sample space to the detector strongly depends on objectives 2 and 3. Larger numerical aperture objectives support larger overlap of light emission cones between the two objectives, and increase the transmission efficiency when traversing those two objectives (Fig. 1b; Supplementary Table 1; “Methods”). Large NA objectives typically have one of two shortcomings that do not fit the SCAPE design: they have short working distances, and thus sterically hinder the turn within the SCAPE coupling design; or they have large magnifications, and thus only capture a small field-of-view. One solution to simultaneously achieve high NA, long working distances, and large fields-of-view is the use of attachments of high index material to objective 3^9–11^. This solution captured nearly all of the fluorescence from the sample, but could be prone to alignment errors when the solution is built in-house, or could be costly when using custom-manufactured objectives.

Another solution is the use of water immersion objectives, which generally have longer working distances at lower magnification. Such a design would be inferior in light capture capability to the designs using the augmented tertiary objectives, but could be easier to execute; the two objectives could in principle move freely under immersion without needing fine alignment of an augmentation between the two objectives. In addition, the objectives in this design would be commercially established, and meet some cost constrained applications. A design using two water immersion objectives as objectives 2 and 3 and coupling the objectives using a water tank was previously proposed^8^. However, this design fixed both objectives into the water tank and sealed them with O-rings, and thus did not facilitate the micrometer movements needed to align the two objectives.

We designed a new “water-coupled SCAPE” layout that enabled freedom of movement for the final segment of imaging path containing objective 3. We created a water immersion tank with a sealed end fitting the second objective and an open top (Fig. 1a, *bottom*; “Methods”). This large open top surface allowed free alignment of the final objective to the intermediate image after the objective 2 and direct access for filling water, all while using gravity to properly hold the water. These mechanical considerations in turn rotated the optical alignment of the final segment of the imaging path between objective 3 and the camera; this path was now at an angle with the table surface of the imaging table rather than parallel to the table surface as in conventional SCAPE (Fig. 1a, *bottom*). To accommodate this semi-vertical path and enable facile optical alignment, we fixed this final segment of the imaging path to a custom platform driven by heavy-duty manual mechanical stages (“Methods”). Finally, this vertical alignment of the final segment in the optical path also necessitated a 90° rotation in the excitation beam. We instantiated this rotation by adding a turning mirror in the common excitation and emission pathway (Fig. 1a, *bottom*).

Our design specifically employed two 20×/1.0 NA water objectives as objective 2 and 3. The transmission efficiency, or light collection capabilities, of this objective pair is approximately three times that of a SCAPE system using a 20×/0.75 NA and 20×/0.45 NA air coupling objectives, which we will exemplify as the “conventional SCAPE” (Fig. 1b). An existing implementation with a 20×/0.75 NA and 50×/0.75 NA air coupling system^8^ could in theory achieve the same transmission and resolution as our water immersion design, but the high magnification of the last objective in the air design would result in a small field-of-view.

### Water-coupled SCAPE imaged fluorescent beads with high resolution

**Figure 2:**
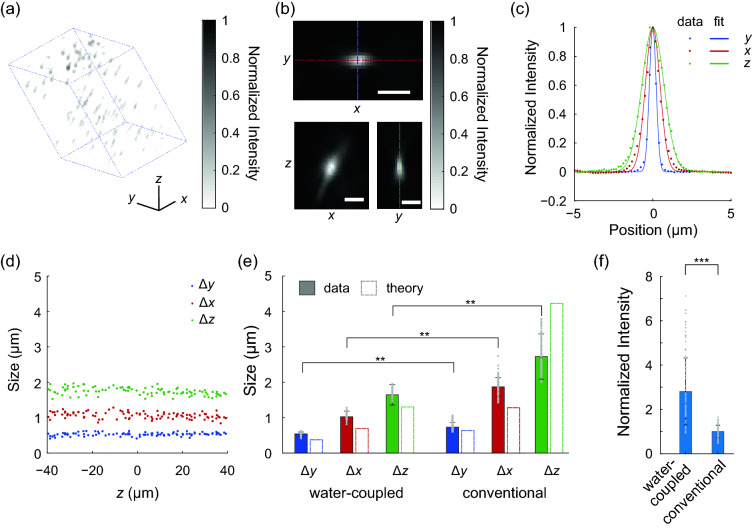
Water-coupled SCAPE resolved fluorescent beads with high resolution and light capture. (**a**) Three-dimensional render of 0.2 µm fluorescent beads embedded in agarose. Scale bars along the axes markers are 30 µm. Blue lines outline the imaged volume. (**b**) Projections of a typical bead along the Cartesian directions. Scale bars are 2 µm. (**c**) Fits of the projections along color-matched dashed lines in (**b**) to Gaussian functions showed that the example bead had FWHMs in the *x*, *y*, and *z* directions of 1.0 µm, 0.52 µm, and 1.6 µm, respectively. (**d**) The fits of beads were relatively constant for beads located at different depths of the sample. (**e**) We compared bead spot FWHMs within 50 µm of the principal imaging plane between the water-coupled and conventional SCAPE platforms. The water-coupled SCAPE significantly outperformed the conventional SCAPE in resolution along all directions. The experimental FWHM most lagged theoretical calculations, except for cases of large Δ*z* that were impacted by the excitation PSF. ** represents *p* < 10^−38^, two-sided Wilcoxon rank-sum test, *n* = 139 beads imaged by water-coupled SCAPE, and 194 beads imaged by conventional SCAPE. (**f**) The water-coupled SCAPE outperformed conventional SCAPE in the intensity of imaged beads. The intensity of both sets of beads imaged by the two microscopes was normalized by the mean intensity of the set of beads imaged by conventional SCAPE. ***Represents *p* < 10^−110^, two-sided Wilcoxon rank-sum test, *n* = 633 beads imaged by water-coupled SCAPE, and 714 beads imaged by conventional SCAPE. All experiments used 1.0 mW at the sample. The distance between planes was 0.062 µm, and the exposure time was 30 ms per layer. Gray points in (**e**) and (**f**) represent data from individual beads, with 100 randomly selected data points shown.

We imaged and visualized the spots of 0.2 µm fluorescent beads embedded in agarose (Fig. 2a; “Methods”). We identified individual beads, calculated their projections along the Cartesian directions (Fig. 2b), and fitted their profiles to Gaussian forms (Fig. 2c). The spot of the oblique light-sheet microscope is anisotropic, which derives from the oblique coupling between objectives 2 and 3. This turn leads to a partial and asymmetric filling of the back aperture of objective 3; the filling of the back aperture in the scan direction was much smaller than in the orthogonal direction, thus leading to an oblong spot. Our water-coupled design, though expanding the range of angles allowed to enter objective 3, retained such anisotropic spots.

Within 50 µm of the principal focal plane, water-coupled SCAPE produced bead spots with full-widths at half-max (FWHMs) along the Cartesian directions as Δ*y* = 0.53 µm ± 0.05 µm, Δ*x* = 1.06 µm ± 0.10 µm, Δ*z* = 1.75 µm ± 0.11 µm (mean ± std, *n* = 120 beads; Fig. 2d,e). These spot sizes were relatively consistent across within a limited range around the principal focal plane; bead sizes in the *x*-, *y*-, and *z*-directions for beads within 20 µm of the principal focal plane were not significantly different from the respective bead sizes for beads between 20 and 50 µm away from the principal focal plane (*p* > 0.45, two-sided Wilcoxon rank-sum test, *n* = 50 beads within 20 µm of the principal focal plane and 70 beads between 20 and 50 µm away from the principal focal plane). The spots sizes along the Cartesian directions were significantly smaller than the spot sizes produced by conventional SCAPE (*p* < 10^−34^, two-sided Wilcoxon rank-sum tests, *n* = 120 beads imaged by water-coupled SCAPE and 194 beads imaged by conventional SCAPE; Fig. 2e). Additional tuning of the excitation light-sheet thickness could further decrease the spot size in the *x*- and *z*-directions (Supplementary Fig. 1), as the oblique excitation light-sheet interacts with the image PSF along multiple Cartesian directions^5^. Carefully alignment of the objective 3-to-camera pathway could trade off spot size in the three Cartesian directions (Supplementary Fig. 2).

We compared our imaging results to theoretical calculations of the 0.2 µm bead spots following earlier frameworks^9^. We totaled the emission that passed from an emitter on the principal focal plane and imaging axis in sample space through all three objectives at the back aperture of objective 3 using ray optics. We then used this weighted back aperture to calculate the emission PSF using Fourier optics (“Methods”). Our experimental resolutions within 50 µm of the focal plane qualitatively matched theoretical calculations of Δ*y* = 0.38 µm, Δ*x* = 0.70 µm, Δ*z* = 1.30 µm, with small aberration caused by off-axis propagation through the objective or aberration away from the principal sample plane causing the residual^7,8^. The conventional SCAPE spot size in the *z*-direction (2.5 µm) was lower than the theoretical spot size in the *z*-direction (4.2 µm), likely because the excitation light-sheet trimmed the PSF in that direction.

We also computed the collection efficiency of water-coupled SCAPE as the integrated intensity of the spot when the spot was most in focus (“Methods”). The mean bead intensity recorded by water-coupled SCAPE was 2.8 times the mean bead intensity recorded by conventional SCAPE, and was significantly larger (*p* < 10^−160^, two-sided Wilcoxon rank-sum test, *n* = 563 beads imaged by water-coupled SCAPE and 638 beads imaged by conventional SCAPE; Fig. 2f). This ratio was in line with the 3.2 ratio expected by theory (Fig. 1b)*.*

### Water-coupled SCAPE revealed detailed substructure within pollen grains

**Figure 3:**
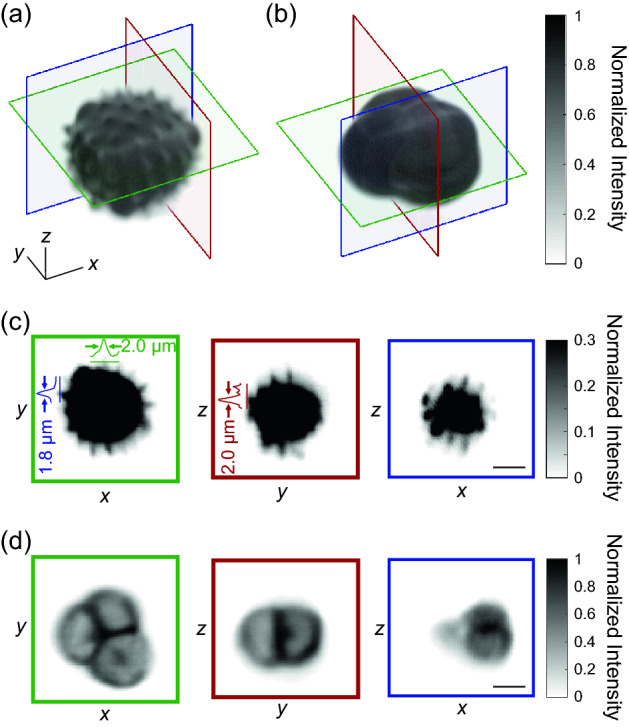
SCAPE imaging of pollen grains showed detailed substructure within each grain. (**a**,**b**) Three-dimensional images of two different pollen grains show different details. The pollen in (**a**) showed small spikes on the surface, while the pollen in (**b**) showed the smooth surface of a multi-grain conglomeration. (**c**) Projections along Cartesian planes were color matched to the planes in (**a**). Each projection showed the sharp, micrometer sized spikes on the surface of the pollen. Color profiles along sharp features are plotted on top of the individual projections. (**d**) Projections along Cartesian planes are color matched to the planes in (**b**). Each projection showed the internal boundaries between the multiple grains. Scale bars and axes all represent 10 µm. The excitation power was 1.0 mW after the excitation objective, the distance between planes was 0.25 µm, and the frame acquisition time was 30 ms.

We imaged the substructure of pollen grains using our microscope. Three dimensional renderings of different types of pollen revealed different substructures (Fig. 3a,b). One type of pollen had sharp spikes on the surface (Fig. 3a), while another type of pollen had a smooth surface (Fig. 3b). Projections along the Cartesian directions revealed similar structure. For the “spiky” pollen, individual spikes appeared clearly in all Cartesian projections (Fig. 3c). The spikes were most clear in the *x–y* plane, because the lateral resolution of SCAPE is sharper than the axial resolution of SCAPE. The “smooth” pollen grain had sufficient transparency to allow imaging of its internal structure. All projections of this pollen grain showed the internal boundaries that delineated the borders between the particles within this conglomeration (Fig. 3d).

### Water-coupled SCAPE imaged neural activity within volumes of the zebrafish brain

We next imaged live larval zebrafish with our microscope. Larval zebrafish is a model organism with high transparency and established toolkits for targeting expression of genetically encoded fluorescent tools to specific cell types^12^. Previous light-sheet microscopes, conventional^13^ or oblique^7,8^, have imaged structure and function within this animal model. We replicate some of these imaging experiments but also show the benefits of using a water-coupled SCAPE with high light collection power.

**Figure 4:**
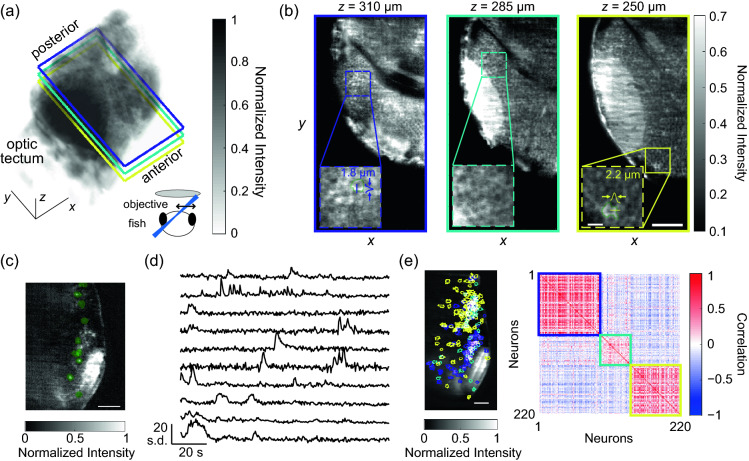
Water-coupled SCAPE revealed sub-cellular structure and dynamic calcium activity of larval zebrafish. (**a**) Three-dimensional rendering of one side of a zebrafish forebrain expressing GCaMP6s revealed the dense connections within the optic tectum. The scale bars along the axes are 50 µm. The volume was obtained with 0.5 µm between layers, and an exposure time of 30 ms per layer. *Bottom right inset*: We show the fish viewed from the frontal direction, along with the relative orientations of the primary imaging objective and excitation light sheet (*blue*). (**b**) Individual planes color matched to the planes marked in (**a**) revealed sub-cellular structure. This zebrafish line localized GCaMP to the extranuclear space, and neurons appeared as rings in the zoomed-in insets. The scale bar in the large images are 50 µm, while the scale bar in the insets are 10 µm. The green outlines in the insets highlight a clear neuron with ring-like expression pattern. The color profiles show the fluorescence intensity along the straight lines; the arrows note the FWHM of the profiled feature. (**c**) A maximum projection at one imaged slice in a zebrafish over two minutes of imaging shows active neurons. Green outlines represent individual neurons found by our analysis pipeline. We obtained these images with 2.6 µm between layers, and an exposure time of 4 ms per layer. (**d**) Activity from heavy green outlines in (**c**) revealed dynamic and independent activity from individual neurons with high peak to background ratio. The volumetric acquisition rate was 10 Hz. The scale bar is 50 µm. (**e**) *Left*: The average of the maximum projections of fluorescence from all layers of the same fish as in (**d**) showed all neurons simultaneously. Outlines showed all neurons from all layers simultaneously. The scale bar is 50 µm. *Right*: The correlation between neurons revealed clusters of neurons that had similar activity, as highlighted by the block diagonal structure of high correlation. The outlines over each block are color matched to the neuron outlines of the left panel. We observed that the cluster of purple neurons localized to the forebrain, and likely resulted from spontaneous visual response. The other two clusters, teal and yellow, localized to the cerebellum and hindbrain.

We imaged larval zebrafish expressing GCaMP6s mounted in agarose (“Methods”). We employed a complementary metal–oxide–semiconductor (CMOS) camera that had configurations for the native resolution or 2 × 2 electronic binning (“Methods”). These two modes could emphasize either resolution or signal fidelity, respectively important for imaging either structure or functional activity. We first examined the structure of zebrafish over volumes as large as 600 µm × 200 µm × 400 µm at the native camera resolution (Fig. 4a). We observed the large distinctive structures of the fish, such as the optic tectum, cerebellum, and hindbrain, as well as small structures such as individual neurons (Fig. 4b).

We next examined the neural activity of individual neurons over a smaller, 600 µm × 100 µm × 300 µm volume at 25 layers per volume and 10 volumetric frames per second using camera binning (Fig. 4c). This volume was more than twice the number of layers and approximately twice the scanned volume examined in previous work^7^; obtaining high SNR throughout this large depth was supported by the high light collection power of water-coupled SCAPE (Supplementary Fig. 3). We employed standard matrix unmixing data analysis procedures to extract individual neurons from the recordings (“Methods”)^14^. We obtained 259 ± 65 neurons for each volume (mean ± std., *n* = 7 volumes from 4 fish). Each neuron displayed prominent transients independently, with rise and decay characteristics typical of calcium activity (Fig. 4d). In recordings that visualized both the forebrain and hindbrain, we hierarchically clustered neurons based on Pearson correlation (Fig. 4e; “Methods”). This clustering revealed functional blocks of neurons that predominantly localized to individual regions of the zebrafish brain; we detected one functional block in the visual responsive area and two functional blocks in the hindbrain.

## Discussion

In this work, we designed, constructed, and characterized a water-coupled SCAPE microscope that coupled two objectives under water immersion. As predicted by theory, the use of large NA objectives produced small spot sizes and large transmission efficiencies through the SCAPE design. Our microscope was able to resolve micron or sub-micron features in multiple inanimate and live animal samples. We resolved the substructure of pollen grains with micrometer resolution, and we resolved calcium transients from awake zebrafish.

SCAPE’s ability to rapidly scan and descan planes in biological samples hopes to image a critical regime of high temporal resolution volumetric recordings. Attaining this regime requires the parallel development of not only optics, but also fluorescent sensor technologies and data analysis algorithms. Our microscope continues the optical evolution of the SCAPE design by allowing the use of water-immersion objectives for both of the last two objectives. Furthermore, our design rotated the portion of the emission path beginning with the third objective. This rotation, and related rotations of the excitation and emission paths, allowed the third objective to dip into the coupling tank. This configuration could support facile alignment by allowing the third objective to freely move within the coupling tank. The use of water immersion objectives also yields the additional benefit of supporting an entirely symmetric remote focusing pathway between objectives 1 and 2. Matching the immersion media of the sample and the immersion media of the oblique objective coupling enables a one-to-one magnification between the sample space and the intermediate space. This symmetry bypasses some of the spherical aberrations that could decrease resolution, as remote focusing systems with unity magnification can exactly achieve the Herschel and Sine imaging conditions when using matching commercial relay lenses with discrete focal lengths^17^. Such symmetry also frees the design of lenses in the remote focusing pathway. Optical engineers could choose from an assortment of focal length ratios that fit in the apertures of the optical pathway, without needing to meet fixed ratios of focal lengths meant to simultaneously meet the Sine and Herschel conditions in a remote focusing module that faces different media on its two ends.

Optical evolution can continue forward with additional engineering of various elements in the microscope’s pathway. First, additional objective development could enhance SCAPE capabilities. Even with our use of large NA objectives, the NAs of objectives 2 and 3 are potential limiters of transmission in SCAPE. Additional custom development of high NA, long-working distance, low-magnification for either water-immersion or oil-immersion objectives should enlarge the capture cones of the last objectives in the SCAPE design. The optical engineering of such objectives could borrow from recent customization of objectives for two-photon microscopy^15,16^. Our rotated emission path design could also be adapted to designs that augment the tertiary objective^9^ while simplifying the requirement to hold the immersion media on the third objective.

Our water-coupled oblique light-sheet microscope slightly underperformed in resolution and light transmission compared to theory. The underperformance in resolution likely arose from residual aberrations of the lenses in the optical pathway. The overall remote focusing pathway from objective 1 to objective 2 requires four lenses and two objectives. Compound spherical aberrations could result in larger spot sizes than theory^17^. It was possible to recover resolution by reducing the light-sheet thickness. Such a tactic could be applicable for experiments that require only limited axial extent, as the light-sheet would rapidly diverge from the principal focal plane due to its narrow focus. The underperformance in transmission efficiency was at least partially due to the lower transmission efficiency of the 1.0 NA water immersion objective, which was tailored for multiphoton imaging.

Our water-coupled oblique microscope underperforms existing microscopes that employ augmented objectives in resolution and light-collection. These augmented objectives compress the emission light cone; oblique light-sheet microscopes using such optics thus support both high transmission efficiencies and high resolution imaging. Our water-coupled design relies on line-of-sight transmission between the coupling objectives, which induces transmission loss. Nevertheless, our design has applications in experimental settings that value flexibility and price points. First, our water-coupling could potentially employ any water immersion objectives that have sufficient working distance by adjusting the o-ring enclosure size. Such flexibility could allow the experimentalist to rapidly change between magnifications and fields-of-view to accommodate different types of samples. Designs that employ augmented objectives would need a custom design for the augmentation or even the entire objective to vary these experimental settings. Second, our water-coupling employs commoditized objectives that, at the moment, cost less than some of the augmented objectives^10^. This price difference could be important in cost sensitive applications.

The continued development of camera technologies could aid the deployment of SCAPE. One general limitation of the light-sheet microscope is the trade-off between the field-of-view, speed, and noise. A camera’s minimum pixel readout time constrains the number of camera rows that can be imaged in a fixed time, which in turn limits the light-sheet microscope’s field-of-view or volumetric imaging rate. Cameras with large pixels could increase field-of-view at equal speeds, but have low spatial sampling and thus low spatial resolution. The CMOS camera used in our implementation included modes that could electronically bin photons in 2 × 2 blocks, but had shorter pixel readout times than existing charge-coupled device (CCD) or electron multiplying CCD (EMCCD) cameras. This binning allowed us to shift between modes that either had large fields-of-view and fast frame speeds when using binning, or had high resolution but moderate frame speeds when not using binning. This binning also bypassed electronic noise associated with digital binning, which reads all individual pixels and thus compounds electronic read noise from each pixel. Because the binning feature is a classic feature of CCD and EMCCD cameras, further development of CCD camera speeds^18^ alongside the development of our hybrid CMOS camera architecture could increase some product of the field-of-view, speed, and image fidelity. In measurements with high photon flux, where shot noise dominates camera electronic noise, CCD or CMOS architectures may offer lower noise floors than EMCCD cameras or intensified cameras that add gain noise^19,20^.

Although photoelectron gain can improve image quality when shot noise is comparable to electronic noise, such situations highlight the necessity of developing brighter fluorescent probes that can outcompete electronic noise during high frame rate imaging of structure or function. Examples of fast structural imaging can attain over 100 volumetric frames per second, such as when imaging large volumes of fixed samples or mechanical motions within small model organisms^8^. These experiments take advantage of the ongoing development of bright fluorescent protein^21^ or dye markers^22^. Imaging neural activity is more challenging due to properties of sensors during dynamic imaging. Millisecond timescale experiments, such as fluorescent voltage imaging, likely require brighter sensors that have low shot-noise even when accessed infrequently during volumetric scanning. The development of voltage indicators with bright fluorescent proteins^23^ or hybridized with bright dyes^24^ has trended in this direction. Such bright sensors will be critical for existing matrix unmixing^14,25^ and deep learning^26,27^ algorithms to accurately segment active neurons. Still, such software packages need additional development of functionalities that match neurons across planes while maintaining the localization of putative sources in space. These procedures highlight the need for experimentalists to employ the various implementations of SCAPE in numerous experimental settings imaging inanimate or live samples, such that a well-rounded training data set can train a robust algorithm that processes volumetric SCAPE data with speed and accuracy.

Because our water-coupled SCAPE design adds to the flexibility of the oblique light-sheet design, we expect that it will serve at least niche applications similar to the ones described above. This iteration of SCAPE provides additional options that integrate with the capability of fluorescent sensors and data analysis pipelines in biological imaging applications.

## Methods

### Microscope setup

We illuminated all samples with a 488 nm laser diode (GH04850B2G, Sharp), driven by a Thorlabs laser driver (LTC56B; Thorlabs). The excitation path collimated and delivered the laser to the sample. We initially magnified the excitation beam size by a factor of 8 using a lens relay pair. We then passed the beam first through an adjustable aperture (VA100, Thorlabs) that controlled the width of the light sheet, then a 100 mm focal length cylindrical lens (Thorlabs LJ1567RM-A), and another adjustable aperture that controlled the thickness of the light-sheet. The beam then passed through two imaging relays that used four achromatic lenses with *f* = 100 mm, 100 mm, 100 mm, and 150 mm (47-641, 47-643; Edmund Optics) in series, all placed in 4*f* conditions. We placed a dichroic mirror (Di03-R488-t3-25 × 36, Semrock) approximately one focal length behind the first lens of the first relay, and a galvo mirror (6240H, Cambridge) approximately one focal length behind the second lens of the first relay. The excitation beam finally passed through the main 20×/0.95 NA water immersion objective (XLUMPLFLN, Olympus).

The emission path initially overlapped with the excitation path back through the same galvo and through the dichroic mirror. The emission then passed through a 150 mm focal length lens (47-643; Edmund Optics) and through a 20×/1.0 NA water immersion objective (XLUMPLFLN, Olympus). The final segment of the emission path was vertically tilted from the end of the second water immersion objective by 38°. The final imaging path consisted of another 20×/1.0 NA water immersion objective (XLUMPLFLN, Olympus), a 300 mm focal length lens (LA1484-A, Thorlabs), and the imaging camera (DaVinci2k, SciMeasure; or Flash4.0V2, Hamamatsu). We aligned and fixed all components of the final imaging path on a custom aluminum breadboard frame and optimized their positions; we then placed the entire assembly on top of three manual mechanical stages (UMR12.63, SM-50, SM-25; Newport) and fixed them together with two L-brackets (AP90RL, Thorlabs). A water immersion tank coupled the final two objectives. This tank was a custom 3D printed holder approximately 50 mm wide, 50 mm deep, and 150 mm long. The 50 mm × 150 mm opening at the top of the tank allowed the optical path containing the third objective to freely move and align with the intermediate image. The end 50 mm × 50 mm surface contained a 35 mm hole, and the rim of the hole was joined with a rubber O-ring (9557K486, McMaster), which fit one of the objectives. We filled the tank with deionized water, and supported the entire tank and objective coupling by a small platform from below.

We constructed the conventional SCAPE with a nearly identical setup as described for the water-coupled SCAPE, save for two differences. First, we replaced the two water immersion objectives 2 and 3 with two air objectives, 20×/0.75 NA (UPLSAPO20×, Olympus) and 20×/0.45 NA (LUCPlanFLN20 × , Olympus), respectively. We also removed the water immersion tank. Second, we replaced the last *f* = 150 mm lens in the emission path with an *f* = 115 mm achromatic lens (75-648, Edmund). This configuration matched the lateral and axial magnification between the sample and the intermediate image between objectives 2 and 3. In order to directly compare the two versions of the microscope with water-coupling and air-coupling, we used the same excitation pathway and objective 1. When comparing bead spots from the air-coupled and water-coupled microscopes, we illuminated the sample with the same excitation power when testing both microscopes.

Our alignment procedures first constructed the excitation pathway. We started with a collimated excitation beam. We iteratively added new lenses and either removed or replaced aligned lenses such that there were even numbers of lenses in the test path, while ensuring the output beam was collimated at the end of the excitation pathway and that the beam passed through two fixed apertures at the microscope output. When aligning the system with the galvo mirror in conjugate space, we measured the distance from the neighboring lenses to the galvo and made sure that the excitation beam was focused on the galvo when there was an odd number of lenses in the excitation pathway. We then added the *f* = 100 mm collimation lens and paired cylindrical lens in front of the dichroic one at a time, with objective 1 removed or in place, respectively, to maintain the collimation of the laser. Finally, we displaced the mirror before this lens pair with a one-dimensional stage (XR25P, Thorlabs) and the associated slit that controlled the light-sheet thickness. This process converted the vertical excitation sheet into an oblique light-sheet. We aligned the emission pathway by illuminating the primary objective with a 532 nm laser source (CPS532, Thorlabs), and collimated the laser emission through objective 1, tube lens, and the paired scan lenses, which were shared between the excitation and emission pathways. We then added the corresponding tube lens and objective 2 to complete the remote focusing arm of the emission pathway. In each of these alignment steps for the emission pathway, we again ensured that there was an even number of lenses in the pathway to collimate the beam by either placing or removing objective 1 in the pathway, and having the test beam pass through two additional fixed apertures after the objective 2 position. We then attached the water tank module to the secondary objective and supported it with a mechanical stage from below. We constructed the emission pathway from the tertiary objective to the camera on a fixed breadboard. We positioned the tertiary objective, tube lens, and camera so that they would be at the designed oblique angle with the optical table surface once the breadboard was erected. We then attached the breadboard to our heavy duty mechanical stages. We finally adjusted the position of this tertiary objective path until the camera imaging plane matched the excitation light-sheet plane, and that the center of the camera matched the focus of the excitation light-sheet.

Our main camera had large 15 µm pixels representing 0.45 µm in sample space; it also supported electronic binning of 2 × 2 pixel blocks, reading the total photons of the block with only one read. These features emphasized field-of-view, frame speed, and the photon to read-noise ratio when using binning, and emphasized resolution when not using binning. In our experiments examining the structure of pollen and zebrafish, we did not use binning and increased the frame exposure time. In our experiments imaging calcium activity in zebrafish, we used binning and increased the frame rate. Although such large pixels are useful for large field-of-view experiments, they could not detail the sub-micrometer PSFs predicted for our microscopes. For bead imaging, we used the scientific CMOS camera, which had 6.5 µm pixels.

During imaging, we used a function generator to drive the galvo mirror with a triangular ramp signal that had a 96–4% duty cycle. We controlled the amplitude and frequency of this signal to determine the distance between planes in the sample volume. We recorded the galvo position and camera frame initialization signals with a digital acquisition board (NI-6361, National Instruments).

### Theoretical estimate of transmission efficiency and resolution

We estimated the theoretical transmission efficiency using geometric optics. We used a point source that emitted a discretized set of rays into all solid angles. Each ray on the unit sphere was defined in two coordinate systems *r*_obj2_ = (*x*_2_, *y*_2_, *z*_2_) and *r*_obj3_ = (*x*_3_, *y*_3_, *z*_3_); one system aligned its *z*-axis with the optical axis of objective 2, while the other system aligned its *z*-axis with the optical axis of objective 3. The two coordinates are related by a three-dimensional rotation matrix, *R*(*α*), where *α* was the defined angle between the optical axes of objectives 2 and 3, such that *r*_obj3_ = *R*(*α*)*r*_obj2_. If needed when using augmented objectives, we first transformed the ray for objective 2 using Snell’s law at the augmentation’s surface and then applied the rotation. We then calculated the azimuthal angle of each ray in each coordinate system as:

1 $${\theta }_{i}=\mathrm{arctan}\frac{\sqrt{{x}_{i}^{2}+{y}_{i}^{2}}}{{z}_{i}},$$

where *i* = 2 or 3 are the same as the coordinates for the reference frames respectively aligned with objectives 2 and 3. We retained all rays that fell within the collection cone of each objective using:

2 $$\left|{\theta }_{i}\right|<{\theta }_{\mathrm{obj}(i)}=\mathrm{arcsin}\frac{{\mathrm{NA}}_{i}}{{n}_{i}},$$

where *θ*_obj(*i*)_ was the cutoff angle for objective *i*, NA_*i*_ was the numerical aperture of objective *i*, and *n*_*i*_ was the index of refraction of the objective *i*’s immersion media. The theoretical transmission efficiency was the ratio of collective emission that met the condition for both objectives 2 and 3 to the collective emission that met the condition for objective 2.

The set of rays collected by objective 3 also helped to define the emission path PSF. We aggregated this set of rays by their location on the back aperture of objective 3 into a histogram. This histogram defined the weighted aperture used for imaging. We then applied the Fourier optics convolution integral in the paraxial approximation to convert this weighted aperture into the PSF. We calculated the spot shape of fluorescent beads by convolving the theoretical PSF with the bead’s dimensions. Finally, we found the theoretical spot size of the beads by first locating the local maximum of the spot, and then computing the intensity profiles along the *x*, *y*, and *z* directions from the local maximum. We fit the resulting profiles to Gaussian functions, and reported the FWHM of these fits as the PSFs along the 3 Cartesian directions.

### Bead and pollen sample preparation

We made the bead samples by diluting a 0.2 µm (24050, Polysciences) bead solution at 1:10,000 in a solution of deionized water and 1.0% low melting point agarose (70050, IBI Scientific) by weight. We placed 20 µL of the mixture on top of a thick microscope slide and waited 2 min until the agarose solidified before imaging.

The pollen sample was a mixed pollen grain slide that embedded multiple types of pollen in mounting media (304264, Carolina Biological Supply Company). We placed all samples on a holder supported by a manual *x-y-z* stage. We imaged all specimens at room temperature. Experimental procedures were carried out in accordance with the institutional and national guidelines and legislation for using plant material.

### Zebrafish preparation

The Institutional Animal Care and Use Committee (IACUC) at Duke University approved all animal experiments. All experiments were performed in accordance with approved protocols, guidelines, and regulations. These experiments were also carried out in compliance with the ARRIVE guidelines. We imaged albino larval zebrafish expressing a GCaMP6s tagged with a nuclear export sequence under the huc promoter at 5–7 days post fertilization using the Tol2kit system^12^. We embedded the fish within the same low melting point agarose gel as above, at 1.5% by weight in egg water, and then drew the fish and agarose inside a glass capillary with an inner diameter of 1.0 mm. After the agarose gel solidified, we extruded the zebrafish body from the capillary while simultaneously removing excess agarose at the fish head to allow for breathing. We then mounted the capillary horizontally inside a 3D printed water tank filled with egg water.

### Data analysis

We linearly interpolated each frame of the imaging movie to fixed planes of the sample volume using the initialization time of each frame and the galvo position recorded by the digital acquisition system. We generated volumetric visualizations of the data by first applying a shear to the stacks of images associated with each volume. We calculated the PSF size by first locating beads as local maxima within the imaging volume. We then calculated the fluorescence intensity profiles of these putative beads along the *x*, *y*, and *z* directions from the local maximum. We then background subtracted the intensities to generate intensity profiles *I*(*w*), where *w* was the *x*, *y*, or *z* coordinate. We fit these profiles to Gaussian functions:

3 $$I\left(w\right)=A{e}^{-\frac{{\left(w-{w}_{0}\right)}^{2}}{2{\sigma }_{w}^{2}}},$$

where *A* was the bead amplitude, *w*_0_ was the center coordinate of the profile, and *σ*_*w*_ was the standard deviation of the profile. We reported the FWHMs of these fits, or *σ*_*w*_ $\sqrt{8\mathrm{log}2}$ ≈ 2.4*σ*_*w*_, as the PSFs along the 3 Cartesian directions.

We defined the *x–y* plane that contained the local maximum of each bead as the principal plane of the bead; we calculated the intensity of the bead by integrating the parameterized *x*- and *y*-fits in this plane. We excluded outlier brightnesses below the 5th percentile and above the 95th percentile in this analysis.

We located putative neurons from the imaging movies using the suite2p pipeline and customized software. We first registered each layer of the imaging movie using suite2p’s one-photon options. We then extracted candidate neurons expected to have a time constant of 1.5 s. Finally, we manually screened the candidate neurons by visualizing the neuron’s shape and activation patterns when the fluorescence of the neuron was high. We calculated the traces from individual neurons by subtracting suite2p’s neuropil signal from the neuron signal for each neuron. We calculated the standard deviation as the quantile-based standard deviation from the lower percentiles of the fluorescence trace, which avoided counting calcium transients as part of the noise.

We used hierarchical clustering to identify groups of neurons with correlated activity. We calculated the similarity between neuron activity traces using the Pearson correlation coefficient (*r*), and subsequently calculated the distance between neuron activity profiles as 1 − *r*. We then created a binary tree that iteratively clustered neurons. Each iteration combined the two clusters with the smallest average linkage distance between clusters. The average linkage distance between two clusters of neurons was the average of the distances between all pairs of neurons where one element was from one cluster, and the other element was from the other cluster.

## Supplementary Information

Supplementary Information.

## Data Availability

Data for generation of figures will be available at Figshare upon the acceptance of the manuscript.
