# Supplementary material for: A fully water coupled oblique light-sheet microscope
Source: Sci Rep. 2022 Apr 8;12:5940. doi: 10.1038/s41598-022-09975-3 (PMC8993908; doi:10.1038/s41598-022-09975-3)
Supplement: Supplementary file 1 — Supplementary Information. [file 41598_2022_9975_MOESM1_ESM.pdf]

Supplementary Information

A fully water coupled oblique light-sheet microscope

Yiyang Gong*, Yuqi Tian, and Casey Baker

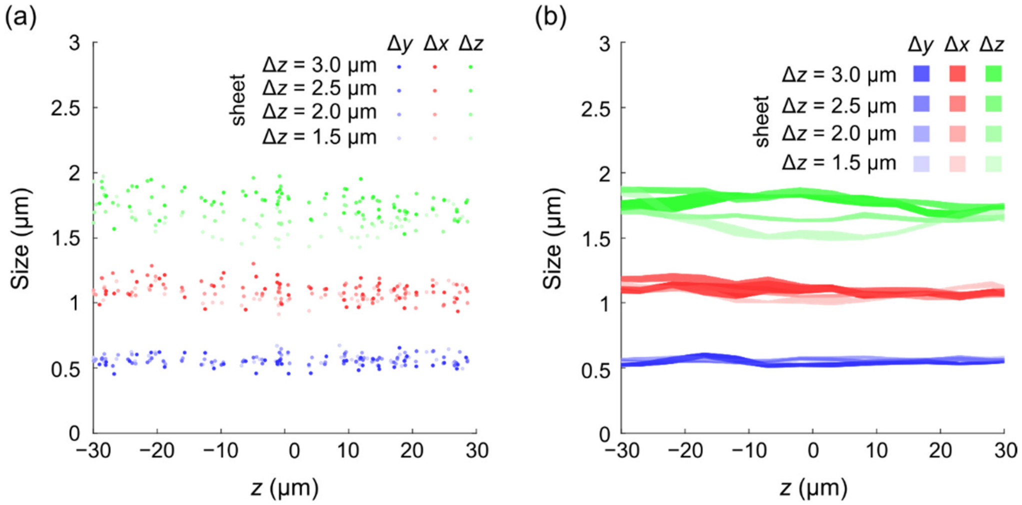

Supplementary Figure 1 – Adjustment to the excitation light-sheet thickness can reduce the spot size near the focal plane. We tuned the light-sheet thickness by adjust the corresponding slit in the excitation path. We show (a) the individual bead spot sizes and (b) spot sizes binned to 10 μm bins along the z-axis in a rolling average. Both measures decreased at the principal focal plane with decreasing excitation width. The *x*- and *z*- spot sizes were impacted by the light-sheet thickness. We tracked the same beads over multiple scans of the same volume using light-sheets of different thicknesses.

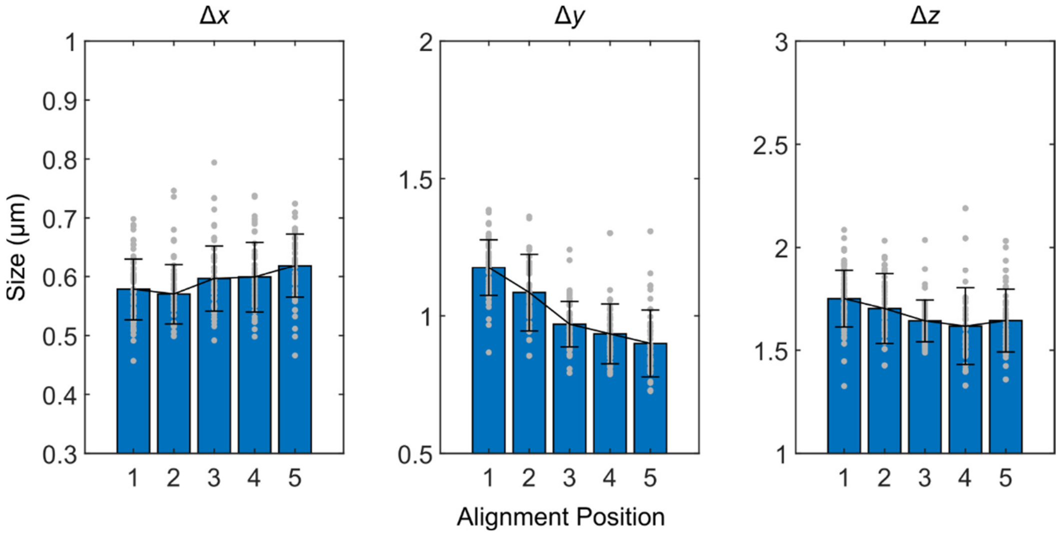

Supplementary Figure 2 – Minor adjustments to the alignment could potentially change the bead spot. We modulated the distance between the secondary and tertiary objectives using a manual stage that guided the objective 3-to-camera portion of the microscope through a range of alignment positions; these position changed the distance between objective 2 and 3 along the optical axis of objective 2. These displacements were paired with minor displacements of the sample while maintaining the same beads in the field of view. We observed that the bead spot size could trade-off resolution along the *x*- and *y*-directions, and the aspect ratio of the bead spot can be tuned. An optimal alignment at position 3 selected an intermediate spot size in both the *x*- and *y*-directions. Dots represent beads spot sizes for beads that were tracked between all experiments. Error bars represent standard deviations.

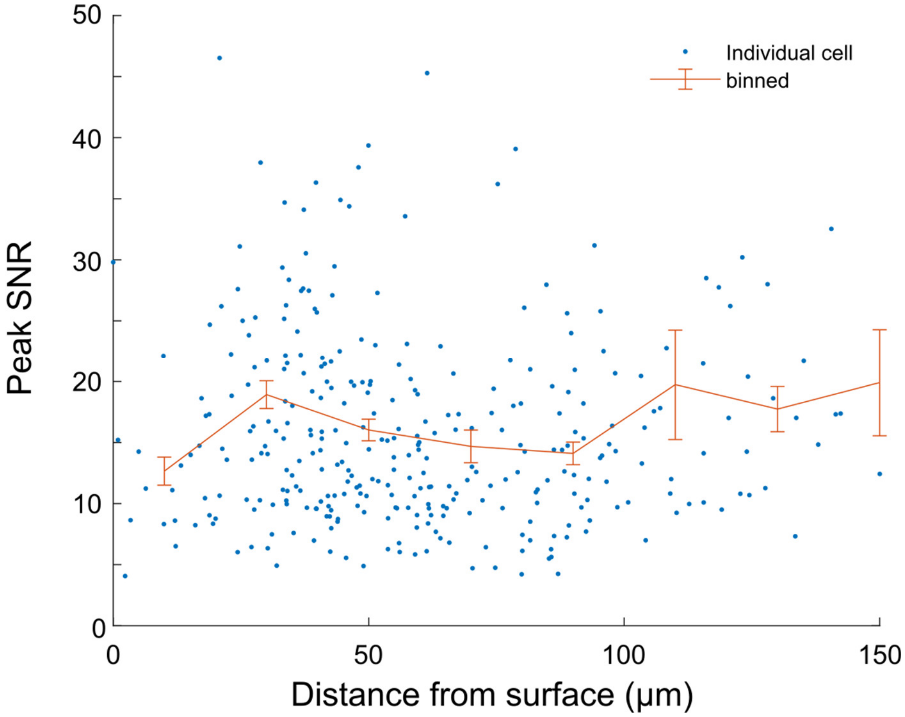

Supplementary Figure 3 – The peak SNR of neurons was approximately constant over different depths. We binned the SNR of individual neurons (blue dots) into bins of 25 μm depth. Error bars are s.e.m.

Supplementary Table 1 – Theoretical spot sizes, transmission efficiencies, and field of view could match specific imaging applications.

**Objective 2 - Objective 3 coupling**	**Δ*x***	**Δ*y***	**Δ*z***	**Transmission efficiency**	**Magnification at intermediate plane***	**Field of view ** **
20×/0.75 NA - 20×/0.45 NA [1]	1.28	0.64	4.23	12%	1.33	1.00 mm
20×/0.75 NA - 20×/0.6 NA [1]	0.92	0.47	2.23	23%	1.33	1.00 mm
20×/1.0 NA (water) - 20×/1.0 NA (water) **(this work)**	0.70	0.37	1.30	38%	1.00	1.10 mm
100×/0.9 NA - 60×/1.0 NA (water) [2]	0.39	0.33	1.21	97%	1.33	0.40 mm

* This calculation assumed the use of a water immersion primary imaging objective to perform experiments similar to the ones in this work.

** The field of view was calculated by using the field number of objective 3 and published magnification of the systems.

Supplementary references

1. Voleti, V., K.B. Patel, W. Li, et al., *Real-time volumetric microscopy of in vivo dynamics and large-scale samples with SCAPE 2.0.* Nat Methods, 2019. **16**(10): p. 1054-1062.
2. Yang, B., X. Chen, Y. Wang, et al., *Epi-illumination SPIM for volumetric imaging with high spatial-temporal resolution.* Nat Methods, 2019. **16**(6): p. 501-504.
